# Seasonal variations in the occurrence of preeclampsia and potential implication of upper respiratory infections in South Korea

**DOI:** 10.1038/s41598-022-14942-z

**Published:** 2022-06-24

**Authors:** Eui Hyeok Kim, Sang Ah Lee, Seunggi Min, Yong Wook Jung

**Affiliations:** 1 Department of Obstetrics and Gynecology, National Health Insurance Service Ilsan Hospital, Goyang-si, Republic of Korea; 2 Korea Research and Analysis Team, National Health Insurance Service Ilsan Hospital, Goyang-si, Republic of Korea; 3 Department of Obstetrics and Gynecology, CHA Gangnam Medical Center, CHA University, 566, Nonhyeon-ro, Gangnam-gu, Seoul, 06135 Republic of Korea

**Keywords:** Urogenital reproductive disorders, Epidemiology

## Abstract

The aim of this study was to examine the effect of seasonal changes on the incidence of preeclampsia (PE) in South Korea and East Asian populations, and to evaluate the relationship between upper respiratory infection (URI) during pregnancy and the development of PE. This cohort study included women who had singleton births between 2012 and 2018 in South Korea. A total of 548,080 first singleton births were analyzed, and 9311 patients (1.70%) were diagnosed with PE. Multivariate analysis showed that older age (≥ 30 years old), low income, residing in the southern part of South Korea, history of cigarette smoking, heavy drinking, higher body mass index, hypertension, or diabetes mellitus were risk factors for PE. Univariate analysis showed that URI was associated with the incidence of PE (*P* = 0.0294). However, this association was not statistically significant in the multivariate analysis (aOR 1.01; 95% CI 0.95–1.07). After adjusting for confounding variables, the occurrence of PE was the highest in December (aOR 1.21; 95% CI 1.10–1.34) and lowest in July and August. This study demonstrated that there are seasonal variations in the occurrence of PE in South Korea. Moreover, URI may be associated with the development of PE.

## Introduction

Preeclampsia (PE) is a disorder of pregnancy that affects 2–8% of all pregnant women. PE is characterized by new-onset hypertension (HTN) and proteinuria after 20 weeks’ gestation. It adversely affects various organs, such as the liver, kidney, brain, and lungs. Additionally, severe PE can lead to multiple organ dysfunction, which is responsible for over 76,000 maternal deaths and 500,000 fetal deaths every year^1^. The definitive treatment for PE is delivery of the placenta and the baby. However, despite the serious adverse effects of PE on maternal and fetal health, its cause and pathogenesis are yet to be elucidated.

Several researchers have indicated that environmental factors, such as the socioeconomic status of the mother, maternal obesity, and cigarette smoking during pregnancy, have potential roles in the development of PE^2–4^. In addition to various maternal demographic factors, seasonal changes are known risk factors for the development of PE. In Norway, the prevalence of PE is highest in the winter months and lowest in August^5^. Similarly, in Sweden, the prevalence of PE is lower in summer than in winter^6^. The prevalence of PE increases during the dry season and decreases during the rainy season in Zimbabwe^7^. In addition to seasonal variations, ethnic differences associated with the monthly variations in the incidence of PE have been reported^8^. However, although previous studies on PE have been conducted in various regions and populations, there is little data on the incidence of PE in East Asian populations and South Korea regions.

The cause of seasonal variations in the occurrence of PE remains unknown. In our previous study, we conducted transcriptome analysis using cell-free RNA in amniotic fluid extracted from patients predicted to develop PE^9^. KEGG pathway analysis showed that various immune pathways, such as those of asthma, antigen processing and presentation, and *Staphylococcus aureus* infection, were dysregulated in patients with PE. Generally, seasonal variations are observed in common cold or upper respiratory infection (URI). Several studies have reported the association between respiratory tract infection and PE. A population-based study in Taiwan showed that the prevalence of PE and eclampsia were higher in women with pneumonia during pregnancy than in women without pneumonia^10^. Getahun et al. reported that seasonal influenza vaccination during pregnancy was associated with reduced risk of influenza and PE as well^11^. Considering the findings of our previous study and the seasonal variations in the occurrence of PE, we hypothesized that URI during pregnancy affects the development of PE. Therefore, we performed this study to examine the effect of seasonal changes on the development of PE in South Korea and East Asian populations, and to evaluate the relationship between URI during pregnancy and the development of PE.

## Results

**Figure 1:**
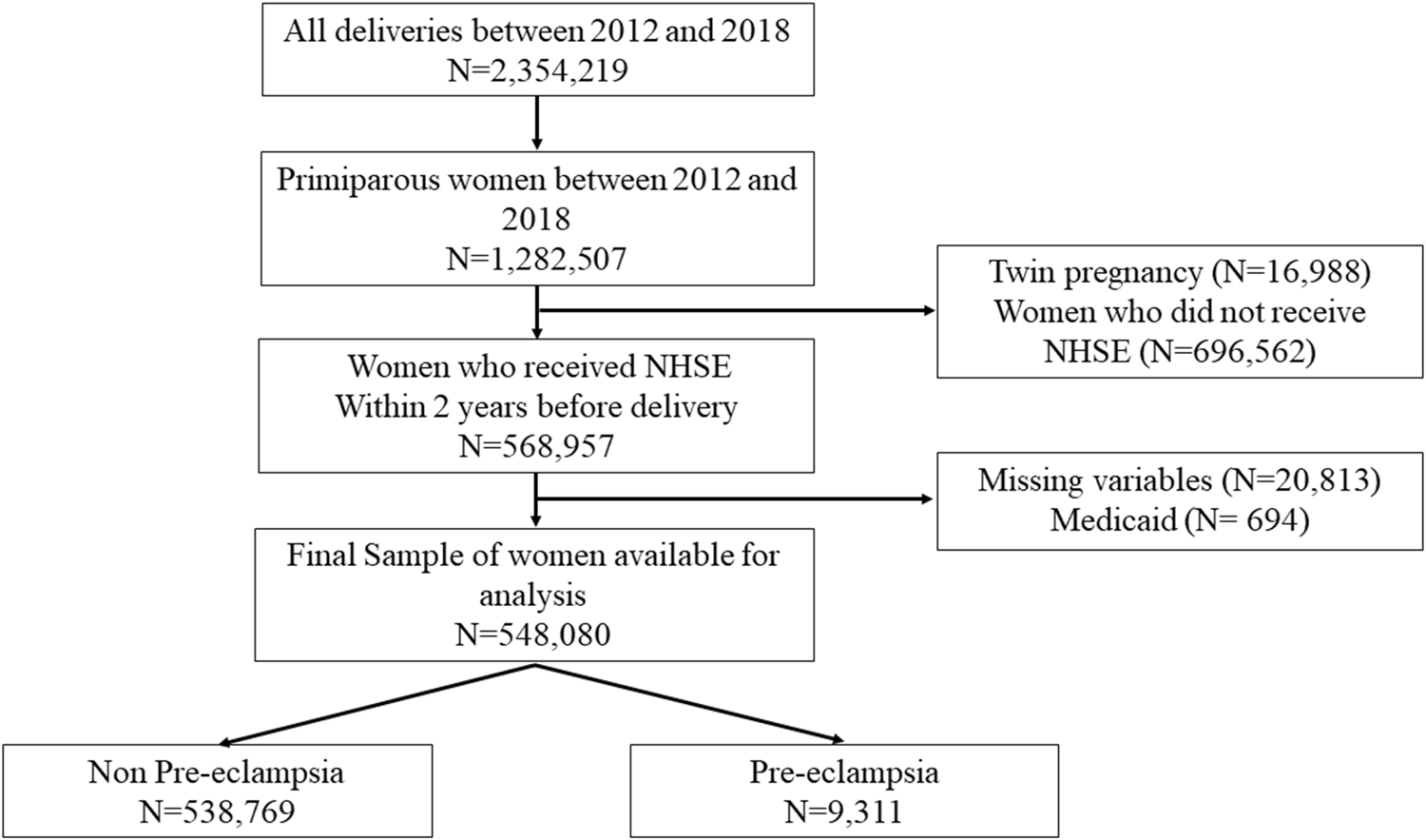
Flowchart of the study.

A total of 2,354,219 births were recorded in Korea between 2012 and 2018 (Fig. 1). Of these, 1,282,507 were in primigravida women. We excluded twin gestations (N = 16,988), women who did not undergo National Health Screening Examination (NHSE) within 2 years before delivery (N = 696,562), those with missing variables (N = 20,813) in the NHSE, and those who were covered by medical aid (N = 694). Therefore, a total of 548,080 singleton deliveries recorded between 2012 and 2018 were included in this study, and PE was diagnosed in 9311 (1.70%) women.

The maternal characteristics of the participants are described in Table 1. In univariate the analysis, age, income, residential area, smoking, physical activity, body mass index (BMI), HTN, diabetes mellitus (DM), and URI were associated with the incidence of PE. In the multivariate logistic regression analysis, all demographic variables were adjusted as possible confounders. The results showed that older age (≥ 30 years), low income, residing in the southern part of South Korea, history of smoking, excessive alcohol consumption higher BMI, HTN, and DM were risk factors for PE. Furthermore, physical activity was associated with the incidence of PE, whereas excessive alcohol consumption was not observed in the univariate analysis. However, heavy drinking was associated with the incidence of PE in the multivariate analysis, whereas physical activity was not. URI was associated with the development of PE in the univariate analysis (*P* = 0.0294); however, multivariate analysis showed that there was no relationship between URI and the prevalence of PE (aOR 1.01; 95% CI 0.95–1.07; *P* = 0.7806). Women aged ≥ 40 years, who were obese, had a history of HTN, or had DM had substantially higher risks for PE ([age ≥ 40 years old: aOR 1.56; 95% CI 1.42–1.72] [obesity: aOR 3.52; 95% CI 3.35–3.69] [HTN: aOR 5.67; 95% CI 5.16–6.24] [DM: aOR 2.56; 95% CI 2.13–3.08]).

**Table 1:** Demographic data of the participants.

Variables	Total	Preeclampsia				
	N (%)	No (N, %)	Yes (N, %)	*P*	aOR (95% CI)	*P*
**Age at the time of delivery**				< 0.0001		
Under 25	12,497 (2.3)	12,282 (98.3)	215 (1.7)		1.12 (0.97–1.29)	0.1221
25–29	138,807 (25.3)	136,839 (98.6)	1,968 (1.4)		1.0	
30–34	290,598 (53.0)	286,117 (98.5)	4,481 (1.5)		1.07 (1.01–1.12)	0.023
35–39	85,663 (15.6)	83,653 (97.7)	2,010 (2.4)		1.45 (1.36–1.54)	< 0.0001
40 or older	20,515 (3.7)	19,878 (96.9)	67 (3.1)		1.56 (1.42–1.72)	< 0.0001
**Income status**				< 0.0001		
1st quartile	65,671 (12.0)	64,394 (98.1)	1,277 (1.9)		1.0	
2nd quartile	161,029 (29.4)	158,051 (98.2)	2978 (1.9)		1.01 (0.95–1.08)	0.7072
3rd quartile	226,536 (41.3)	222,926 (98.4)	3,610 (1.6)		0.93 (0.87–0.99)	0.0271
4th quartile	94,844 (17.3)	93,398 (98.5)	1,446 (1.5)		0.90 (0.83–0.97)	0.0065
**Residential area**				0.0002		
Central	362,046 (66.1)	356,064(98.4)	5,982 (1.7)		1.00	
Southern	186,034 (33.9)	182,705 (98.2)	3,329 (1.8)		1.08 (1.03–1.12)	0.0010
**Cigarette smoker**				< 0.0001		
No	530,431 (96.8)	521,550 (98.3)	8,881 (1.7)		1.00	
Yes	17,649 (3.2)	17,219 (97.6)	430 (2.4)		1.24 (1.12–1.38)	< 0.0001
**Excessive alcohol consumption**				0.1092		
No	445,903 (81.4)	438,388 (98.3)	7,515 (1.7)		1.00	
Yes	102,177 (18.6)	100,381 (98.2)	1,796 (1.8)		1.07 (1.01–1.13)	0.0134
**Physically active**				0.0445		
No	344,318 (62.8)	338,562 (98.3)	5,756 (1.7)		1.00 (0.96–1.04)	0.9669
Yes	203,762 (37.2)	200,207 (98.3)	3,555 (1.7)		1.00	
**BMI**				< 0.0001		
Underweight	69,326 (12.7)	68,760 (99.2)	566 (0.8)		0.70 (0.64–0.76)	< 0.0001
Normal	337,373 (61.6)	333,331 (98.8)	4,042 (1.2)		1.00	
Overweight	70,968 (13.0)	69,511 (98.0)	1,457 (2.1)		1.66 (1.56–1.76)	< 0.0001
Obese	70,413 (12.9)	67,167 (95.4)	3,246 (4.6)		3.52 (3.35–3.69)	< 0.0001
**History of HTN**				< 0.0001		
No	543,382 (99.1)	534,656 (98.4)	8,726 (1.6)		1.00	
Yes	4,698 (0.9)	4,113 (87.6)	585 (12.5)		5.67 (5.16–6.24)	< 0.0001
**History of DM**				< 0.0001		
No	461,062 (84.1)	453,727 (98.4)	7,335 (1.6)		1.00	
Yes	1,355 (0.3)	1,193 (88.0)	162 (12.0)		2.56 (2.13–3.08)	< 0.0001
GDM	85,663 (15.6)	83,849 (97.9)	1,814 (2.1)		1.17 (1.11–1.23)	< 0.0001
**URI during pregnancy**				0.0294		
No	476,485 (86.9)	468,461 (98.3)	8,024 (1.7)		1.00	0.7806
Yes	71,595 (13.1)	70,308 (98.2)	1,287 (1.8)		1.01 (0.95—1.07)	

BMI, body mass index; HTN, hypertension; GDM, gestational diabetes mellitus; URI, common cold.

**Figure 2:**
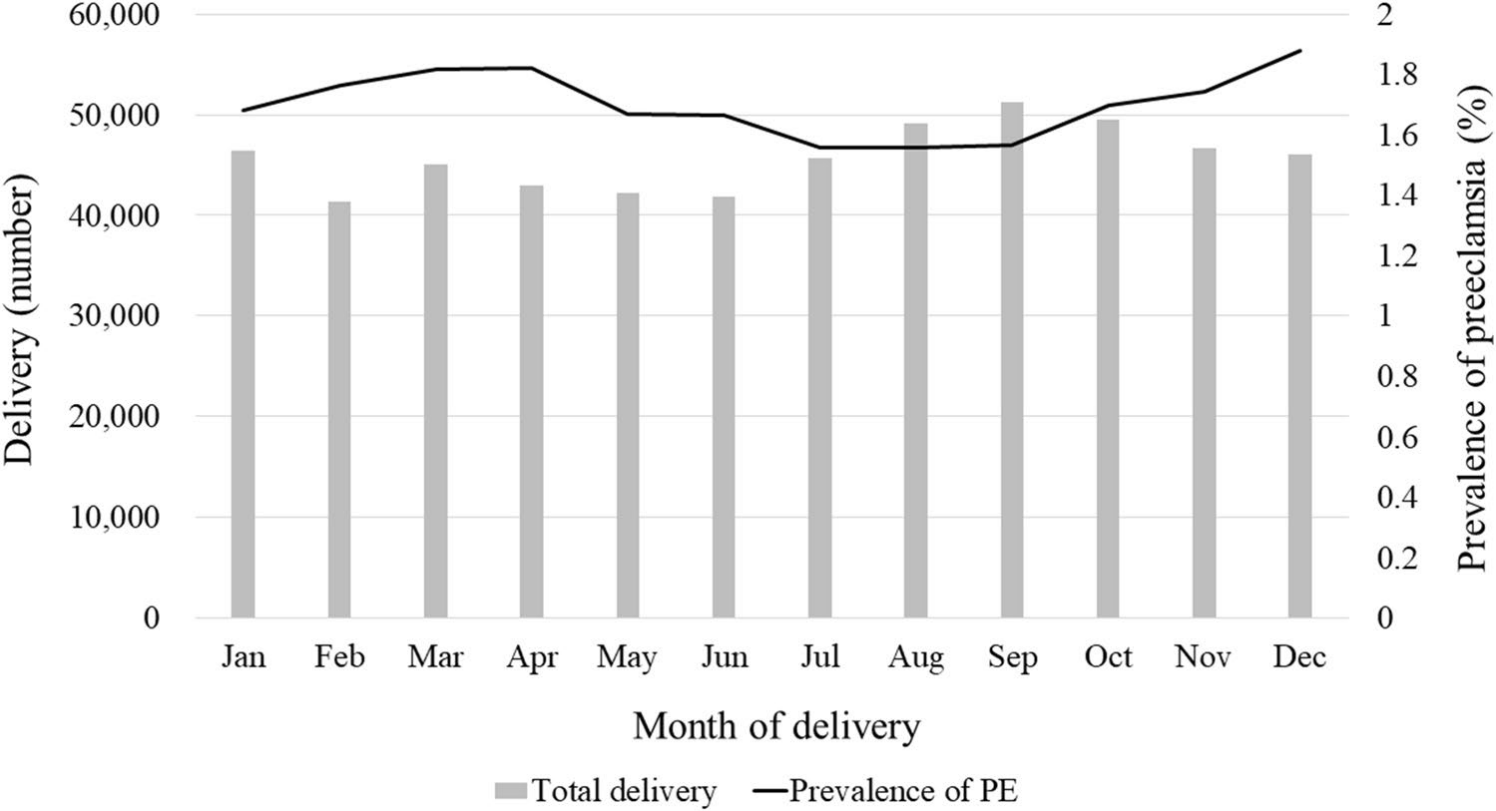
Prevalence of preeclampsia according to the month of delivery.

**Figure 3:**
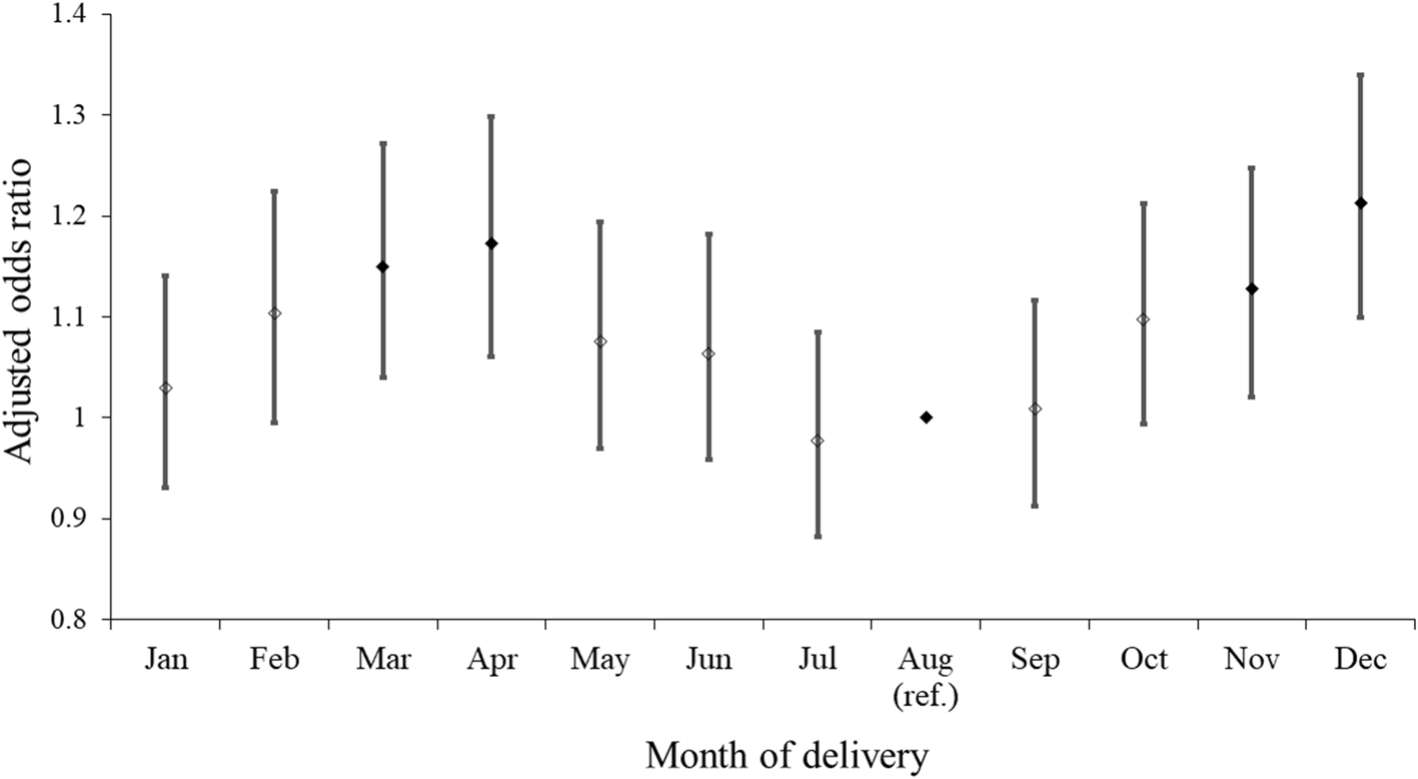
Association between the month of delivery and preeclampsia. *Adjusted for age at the time of delivery, income status, residential area, smoking status, drinking status, physical activity, body mass index, history of hypertension before pregnancy, diabetes mellitus, and upper respiratory infection during pregnancy.

The prevalence of PE was the highest (1.88%) in December and lowest (1.56%) in July and August (Table 2, Fig. 2). After adjusting for confounding variables, the occurrence of PE was still the highest in December (aOR 1.21; 95% CI 1.10–1.34) and lowest in July and August. Figure 3 shows the monthly variations in the occurrence of PE. When stratified according to the season of delivery before adjusting for confounding variables, the prevalence of PE was the lowest in the summer, increased gradually in the fall, and was highest in the spring. Adjustment for demographic variables did not alter seasonal trends in the development of PE. The prevalence ratios of PE were as follows: winter, 1.10 (95% CI 1.04–1.17); spring, 1.12 (95% CI 1.06–1.19); and fall, 1.06 (95% CI 1.00–1.13). The prevalence of PE in spring and winter did not differ.

**Table 2:** Prevalence rates and ratios of preeclampsia according to the month of delivery, with August as the reference month.

Month of delivery	Total N (%)	Preeclampsia		Prevalence	*P*	Unadjusted OR (95% CI)	*P*	Adjusted OR (95% CI)	*P*
		No (N, %)	Yes (N, %)						
January	46,397 (8.5)	45,616 (98.3)	781 (1.7)	1.68	0.0002	1.08 (0.98–1.20)	0.1257	1.03 (0.93–1.14)	0.5640
February	41,351 (7.5)	40,621 (98.2)	730 (1.8)	1.77		1.14 (1.02–1.26)	0.0149	1.10 (1.00–1.22)	0.0609
March	45,053(8.2)	44,235 (98.2)	818 (1.8)	1.82		1.17 (1.06–1.29)	0.0022	1.15 (1.04–1.27)	0.0064
April	42,954 (7.8)	42,172 (98.2)	782 (1.8)	1.82		1.17 (1.06—1.30)	0.0020	1.17 ( 1.06–1.30)	0.0020
May	42,194 (7.7)	41,489 (98.3)	705 (1.7)	1.67		1.07 (0.97–1.19)	0.1775	1.08 (0.97–1.19)	0.1656
June	41,859 (7.6)	41,162 (98.3)	697 (1.7)	1.67		1.07 (0.97–1.19)	0.2012	1.06 (0.96–1.18)	0.2405
July	45,716 (8.3)	45,004 (98.4)	712 (1.6)	1.56		1.00 (0.90–1.11)	0.9927	0.98 (0.88–1.09)	0.6766
August	49,160 (9.0)	48,394 (98.4)	766 (1.6)	1.56		1.00		1.00	
September	51,203 (9.3)	50,402 (98.4)	801 (1.6)	1.56		1.00 (0.91–1.11)	0.9371	1.01 (0.91–1.12)	0.8553
October	49,505 (9.0)	48,664 (98.3)	841 (1.7)	1.70		1.09 (0.99–1.21)	0.0811	1.10 (0.99–1.21)	0.0670
November	46,683 (8.5)	45,869 (98.3)	814 (1.7)	1.74		1.12 (1.01–1.24)	0.0243	1.13 (1.02–1.25)	0.0185
December	46,005 (8.4)	45,141 (98.1)	864 (1.9)	1.88		1.21 (1.10–1.33)	0.0001	1.21 (1.10–1.34)	0.0001
**Delivery season**					0.0002				
Spring	130,201 (23.8)	127,896 (98.2)		1.77		1.12 (1.05–1.18)	0.0003	1.12 (1.06–1.19)	0.0002
Summer	136,735 (25.0)	134,560 (98.4)		1.59		1.00		1.00	
Fall	147,391 (26.9)	144,935 (98.3)		1.67		1.05 (0.99–1.11)	0.1116	1.06 (1.00–1.13)	0.0407
Winter	133,753 (24.4)	131,378 (98.2)		1.78		1.12 (1.06–1.19)	0.0002	1.10 (1.04–1.17)	0.0015

Adjusted for age at the time of delivery, income status, residential area, smoking status, drinking, physical activity, body mass index, history of hypertension before pregnancy, diabetes mellitus, and upper respiratory infection during pregnancy.

## Discussion

In this study, we analyzed the effects of seasonal variations on the incidence of PE in South Korea, and evaluated the relationship between URI during pregnancy and the development of PE. The results showed that several patient demographic factors, including age, socioeconomic status, residential area, habits and behavior, BMI, HTN, and DM were associated with the risk of PE. We also observed seasonal variations in the occurrence of PE in South Korea. In this study cohort, the incidence of PE was the lowest in August but increased steadily from August to December, thus reaching its nadir in spring. In addition, this seasonal trend was maintained after maternal characteristics were adjusted as potential confounders. Furthermore, the results indicated that URI was associated with the occurrence of PE. However, the association between the development of PE and URI was not maintained in the multivariate analysis.

**Figure 4:**
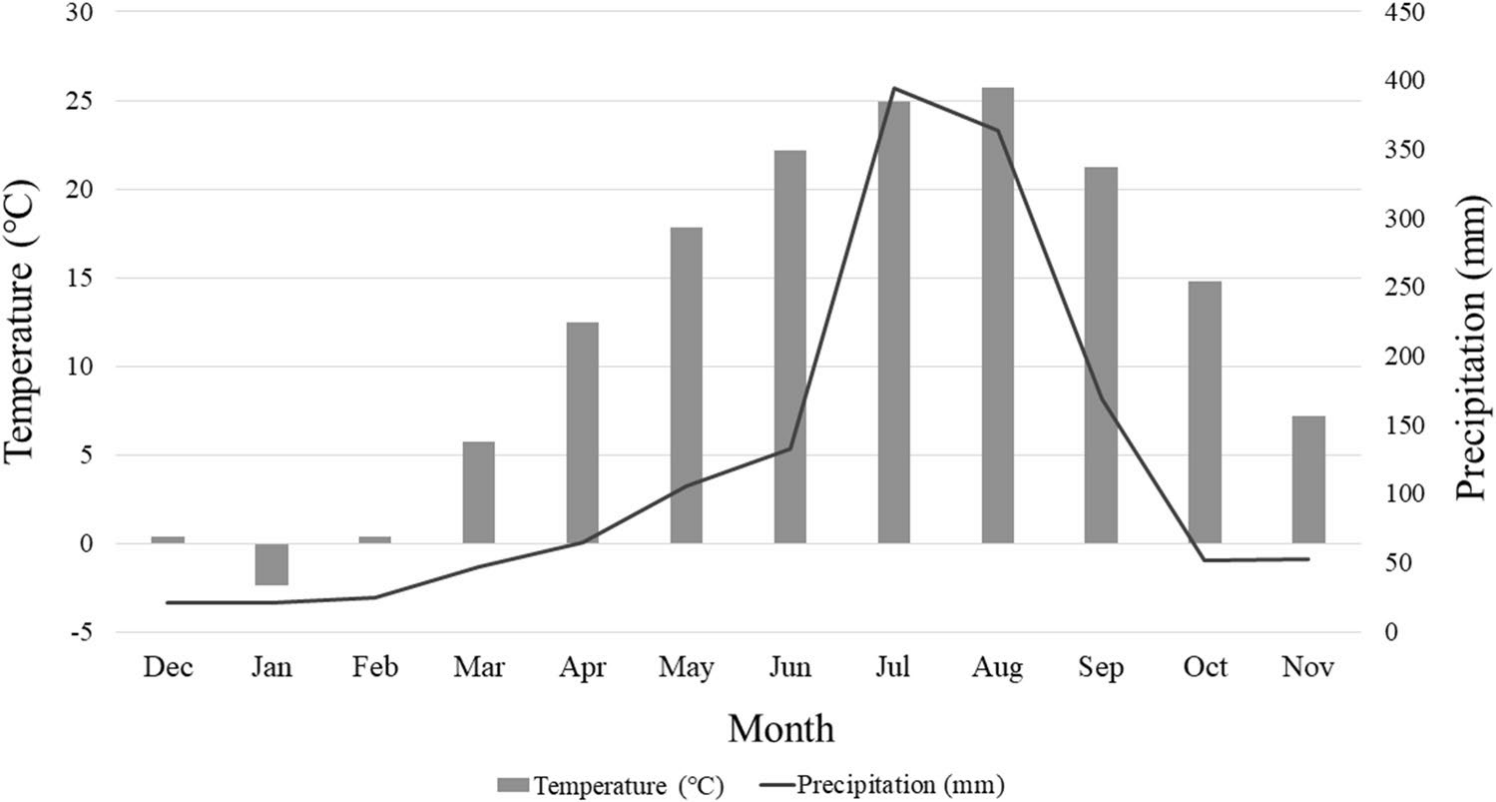
Climate chart of South Korea.

In a retrospective cohort study by Magnus et al., which included 1,869,388 recorded deliveries in Norway within over 30 years (1967–1998), the risk of PE was lowest in August and highest in the winter months^5^. Moreover, several researchers have also noted seasonal trends in the occurrence of PE. Ros et al. reported that the risk of PE was lowest in the summer and among women who delivered outside Nordic countries^6^. Phillips et al. reported that in Vermont, United States, the incidence of PE in the summer was decreased compared to that in spring^12^. In Texas, although minimal seasonal variations were reported, the prevalence of PE was lowest in the fall and highest in the winter^13^. This observation was maintained after adjusting for several confounders. South Korea is located in East Asia and has a temperate climate with four distinct seasons. The mean temperature of Seoul, South Korea’s capital is -4 °C in January and 24.0 °C in August (Fig. 4). Although there were some differences depending on the region, seasonal variations in PE occurrence in the present study are consistent with those of previous studies conducted in various regions.

Differences between the monthly variations in the incidence of PE among white and black women has been reported. Bodnar et al. stated that the incidence of PE among white women in the United States decreased during summer^8^. However, this seasonal pattern was not noted in black women. South Korea is an ethnically homogenous country. Approximately 96% of the total population are of Korean ethnicity and are East Asian; even half of the immigrants in South Korea are from China^14^. Therefore, our data, which is representative of the East Asian population, demonstrates that the incidence of PE in East Asian women varies according to seasons, as in white women.

The prevalence of PE may vary according to seasons. Some researchers have proposed that cold temperatures may cause peripheral vasoconstriction, which increases placental vascular resistance, thus resulting in placental insufficiency and PE. However, there are several limitations to the hypothesis of the relationship between seasonal variation and PE. Seasonal variation in PE exists in regions where there is no winter^7^. In addition, ethnic differences in the monthly variations of the incidence of PE have been reported^8^. These observations suggest that environmental exposures related to monthly changes in lifestyle patterns, such as acute infection, dietary intake, and sunlight exposure, may contribute to the pathogenesis of PE. Of these environmental factors, infections plays a crucial role in the initiation and aggravation of uteroplacental insufficiency^15^. Various infectious diseases activate systemic inflammatory responses and endothelial injury, which may lead to uteroplacental atherosclerosis and placental hypoxia. These responses to inflammation result in an increased risk of PE. Several studies have demonstrated that urinary tract infection and periodontal disease are also potential risk factors for PE^16–19^.

Among other infections, those related to URI vary with seasons. We hypothesized that the immune response associated with URI during pregnancy affects the development of PE. Although multivariate analysis did not show a statistically significant association between the development of PE and URI, univariate analysis demonstrated that URI was associated with an increased prevalence of PE. We observed the effect of changes in seasons on the incidence of PE between November and April. The incidence of PE was lower in January during winter, and the incidence of URI was higher in seasons when the daily temperature difference was large. Interestingly, the mean number of patients with URI within a 5-year period (from 2010 to 2014) was lower in January as was the incidence of PE in South Korea^20^. Thus, the data of the present study show a potential association between URI and PE. A correlation between URI caused by viral infection and increased odds of PE was observed in a prospective study of pregnant women with asthma^21^. Furthermore, Romanyuk et al. observed a statistically significant association between pneumonia and severe PE in their population-based study^22^. However, the results of some studies do not suggest an association between URI and the development of PE. Minassian et al. conducted a population-based case–control study of 1,533 patients with PE and 14,236 randomly selected controls to assess the effect of URI on the risk of PE^18^. The authors excluded any non-specific URI from the analysis, such as acute respiratory infection and respiratory tract infection, but did not observe any association between URI and PE. The conflicting results of these studies may be a result of the varying definitions of URI and PE and the heterogeneity of the study populations.

The present study has several limitations. First, we could not determine exact pregnancy dates, which is important in distinguishing the subtypes of PE and in estimating the month of conception. PE is divided into two subtypes according to disease onset: early (< 34 gestational weeks) and late (≥ 34 gestational weeks). The pathophysiology of these two subtypes differs. Therefore, as we could not determine the subtypes of PE in this study, the effect of seasonal changes on the development of PE according to its subtypes could not be elucidated. Second, we did not analyze PE in relation to the timing of conception. Phillips et al. suggested that the timing of conception is more strongly related to seasonal variations in the incidence of PE than the season of delivery^12^. Third, the timing of infection during pregnancy may affect pregnancy outcomes related to PE. Placental development is complete by the end of the first trimester of pregnancy. Development of URI during this critical period may have a greater impact on the development of PE. However, we could not evaluate the association between the development of PE and the timing of URI during pregnancy because we had limited data on pregnancy dates. Fourth, misclassification bias should be considered when analyzing data from the NHIS claim database, as there might be differences in coding practices according to the type of medical institute. In addition, insurance reimbursement policies affect coding practice of medical institution. To minimize coding bias, we investigated prescribed medications along with ICD-10 codes.

Our study is a retrospective cohort study, which has a significant misclassification bias. Hence, our study cannot provide exact information regarding the relationship between the incidence of PE and URI, neither can it be used to explain why URI and seasonal changes affect the prevalence of PE. Therefore, further prospective studies with more stringent definitions of URI are required to demonstrate the association between PE prevalence and URI. Using animal model of PE would give an insight into the pathogenesis of how URI during pregnancy affects the occurrence of PE.

However, this study has several strengths. To the best of our knowledge, there is little data on the association between seasonal variations and the incidence of PE in East Asian populations and South Korea. Therefore, the present study makes a considerable contribution to existing research. Another strength of this study is the use of a national database. We examined all recorded births between 2012 and 2018 in South Korea; therefore, the study data provides information that is more reliable. Moreover, we investigated the relationship between URI and the occurrence of PE. Several studies have been conducted to evaluate the association between the development of PE and urinary tract infection; however, only few studies have been conducted to investigate the relationship between URI and PE.

In summary, the present study demonstrated that there are seasonal variations in the occurrence of PE in South Korea. In this study cohort, the incidence of PE was the lowest in August but gradually increased from August to December, thus reaching its nadir in spring. In addition, univariate analysis showed that URI was associated with the occurrence of PE. Further studies that use prospectively collected data and exact pregnancy dates to analyze the association between seasonal variations and the development of PE are required to evaluate those factors involved in the seasonal trends observed in the occurrence of PE development. Clarifying the biological mechanisms by which seasonal variations and URI affect the development of PE are also necessary in order to elucidate the pathogenesis of PE.

## Methods

This was a retrospective cohort study conducted using National Health Insurance Service (NHIS) claims data, which were collected from January 2012 to December 2018. The government of South Korea provides universal healthcare coverage for 97% of the population residing in South Korea through the NHIS. The remaining 3% are covered by medical aid to protect them from the financial burden of excessive medical expenditure. As part of the NHIS healthcare program, beneficiaries are invited to participate in a NHSE program biannually. The NHSE consists of health examinations and interviews, including questions regarding patients’ demographic, socioeconomic, and lifestyle characteristics. The results of the NHSE are stored in the NHIS database. To facilitate the evaluation of pre-pregnancy characteristics, only women who underwent an NHSE at least 2 years before their first delivery were included in the analysis. Those with missing data in the database were excluded. This study complies with the principles of the Declaration of Helsinki. The Institutional Review Board of the National Health Insurance Service (NHIS) Ilsan Hospital approved this study (NHIMC 2020). All methods were performed in accordance with relevant guidelines and regulations. Written informed consent was waived by the Institutional Review Board of the National Health Insurance Service Ilsan Hospital because of the large number of participants in the cohort and the retrospective nature of the study.

The pathophysiology of HTN during pregnancy differs between parous and nulliparous women; therefore, only nulliparous women were included in the present study. The women included were identified using International Classification of Diseases, 10th revision (ICD-10) codes O11, O14, and O15 to identify cases of PE. The Korean Society of Obstetrics and Gynecology recommends that PE be diagnosed if gestational HTN and proteinuria are present. Gestational HTN was defined as ≥ 2 systolic blood pressure measurements ≥ 140 mmHg and/or a diastolic blood pressure ≥ 90 mmHg, which was observed for the first time during antenatal care. Proteinuria was defined as a 1 + result on two random urine dipstick tests or a 2 + result on one urine dipstick test.

We examined known demographic risk factors for PE, including maternal age, income status, history of smoking, physical activity, heavy drinking, BMI, and medical history of HTN, DM, or URI during pregnancy. The women were stratified into four groups (quartiles) according to their economic status and according to BMI: underweight (BMI < 18.5 kg/m^2^), normal (18.5–24.9 kg/m^2^), overweight (25–29.9 kg/m^2^), obese (≥ 30 kg/m^2^). Regarding alcohol consumption habits, the included women were categorized as non-heavy or heavy drinkers. Heavy drinkers were defined as those with an alcohol consumption status that needed correction, those who consume alcohol more than four times per week, or those who have more than four drinks at a time. This definition is based on the criteria outlined by the National Institute on Alcohol Abuse and Alcoholism and revised by the Ministry of Health and Welfare (MOHW) in consideration of the alcohol consumption scenario in Korea. Regarding smoking status, the participants were categorized as current or non-smokers based on their NHSE results. For physical activity, the MOHW has presented a physical activity guide for Koreans based on the physical activity guidelines published by the US Department of Health and Human Services. According to the guidelines, physical activity is defined as more than three episodes of high-intensity workouts per week or more than five episodes of intermediate workouts per week.

South Korea has four distinct seasons: spring (March–May), summer (June–August), fall (September–November), and winter (December–February). Winter temperatures are higher along the southern coast (southern region) and considerably lower in the mountainous interior (central region). Therefore, we classified the participants’ areas of residence into southern or central regions. Regarding URI during pregnancy, the following ICD-10 codes were used to define/identify URI: R05, cough; R04, hemorrhage from respiratory passage; A37, whooping cough; J00–J06, acute upper respiratory infection; J10, influenza due to other identified influenza virus; and B34, viral infection of unspecified site.

### Statistical analysis

The demographic characteristics of the PE and control groups were compared using the chi-square test for categorical variables. The prevalence of births complicated by PE in each month and season was calculated. The relative risks for PE according to the month and season of delivery were estimated as adjusted prevalence odds ratios (aORs) using the month with the lowest risk as the reference. To adjust for possible confounding variables, multiple logistic regression was used to analyze the relative risk for PE using the other variables as ORs. The exact delivery date of each woman was identified using the NHIS claims data. The monthly prevalence of PE was calculated by dividing the number of women with PE in a month by the number of deliveries in that month. Statistical analyses were performed using SAS version 9.4 software (SAS Institute, Inc.; Cary, NC, USA).
