# Author Correction: Seasonal variations in the occurrence of preeclampsia and potential implication of upper respiratory infections in South Korea

**DOI:** 10.1038/s41598-022-16500-z

**Published:** 2022-07-27

**Authors:** Eui Hyeok Kim, Sang Ah Lee, Seunggi Min, Yong Wook Jung

**Affiliations:** 1 Department of Obstetrics and Gynecology, National Health Insurance Service Ilsan Hospital, Goyang-si, Republic of Korea; 2 Korea Research and Analysis Team, National Health Insurance Service Ilsan Hospital, Goyang-si, Republic of Korea; 3 Department of Obstetrics and Gynecology, CHA Gangnam Medical Center, CHA University, 566, Nonhyeon-ro, Gangnam-gu, Seoul, 06135 Republic of Korea

Correction to: *Scientific Reports* https://doi.org/10.1038/s41598-022-14942-z, published online 24 June 2022

The original version of this Article contained an error in the spelling of the author Eui Hyeok Kim which was incorrectly given as Eui hyeok Kim.

The original Article has been corrected.

